# B Lymphocyte Stimulator (BLyS) Is Expressed in Human Adipocytes *In Vivo* and Is Related to Obesity but Not to Insulin Resistance

**DOI:** 10.1371/journal.pone.0094282

**Published:** 2014-04-11

**Authors:** Nike Müller, Dominik M. Schulte, Susann Hillebrand, Kathrin Türk, Jochen Hampe, Clemens Schafmayer, Mario Brosch, Witigo von Schönfels, Markus Ahrens, Rainald Zeuner, Johann O. Schröder, Matthias Blüher, Christian Gutschow, Sandra Freitag-Wolf, Marta Stelmach-Mardas, Carina Saggau, Stefan Schreiber, Matthias Laudes

**Affiliations:** 1 Department I of Internal Medicine, University of Kiel, Kiel, Germany; 2 Department of General, Abdominal, Thoracic and Transplantation Surgery, University of Kiel, Kiel, Germany; 3 Department for Internal Medicine, Clinic for Endocrinology and Nephrology, University of Leipzig, Leipzig, Germany; 4 Department of General, Visceral and Cancer Surgery, University of Cologne, Cologne, Germany; 5 Institute of Medical Informatics and Statistics, University of Kiel, Kiel, Germany; McGill University, Canada

## Abstract

Inflammation and metabolism have been shown to be evolutionary linked and increasing evidence exists that pro-inflammatory factors are involved in the pathogenesis of obesity and type 2 diabetes. Until now, most data suggest that within adipose tissue these factors are secreted by cells of the innate immune system, e. g. macrophages. In the present study we demonstrate that *B lymphocyte stimulator* (BLyS) is increased in human obesity. In contrast to several pro-inflammatory factors, we found the source of BLyS in human adipose tissue to be the adipocytes rather than immune cells. In grade 3 obese human subjects, expression of BLyS *in vivo* in adipose tissue is significantly increased (p<0.001). Furthermore, BLyS serum levels are elevated in grade 3 human obesity (862.5+222.0 pg/ml vs. 543.7+60.7 pg/ml in lean controls, p<0.001) and are positively correlated to the BMI (*r* = 0.43, p<0.0002). In the present study, bariatric surgery significantly altered serum BLyS concentrations. In contrast, weight loss due to a very-low-calorie-formula-diet (800 kcal/d) had no such effect. To examine metabolic activity of BLyS, in a translational research approach, insulin sensitivity was measured in human subjects *in vivo* before and after treatment with the human recombinant anti-BLyS antibody belimumab. Since BLyS is known to promote B-cell proliferation and immunoglobulin secretion, the present data suggest that adipocytes of grade 3 obese human subjects are able to activate the adaptive immune system, suggesting that in metabolic inflammation in humans both, innate and adaptive immunity, are of pathophysiological relevance.

## Introduction

Obesity is associated with a reduced life-span [1] and represents a fast-growing health problem that is reaching epidemic proportions worldwide [2]. It leads to several chronic co-morbidities including type 2 diabetes, dyslipidemia and atherosclerosis [3]. The risk to develop type 2 diabetes is estimated to be nine fold higher for obese than for lean men [4].

It is already known that the development of insulin resistance and type 2 diabetes is associated with inflammatory mechanisms in adipose tissue [5]. This relationship can be explained by hypertrophic and functionally impaired adipocytes in visceral and subcutaneous fat depots due to a positive energy balance. In this pathophysiological condition various bioactive molecules are being produced and secreted by adipocytes which can activate the infiltration of cells of the innate immune system, e. g. macrophages. [5]–[7]. These cells are able to inhibit adipogenesis from mesenchymal stem cells via classical pro-inflammatory cytokines and wnt-molecules in a paracrine manner [6]–[8]. As a consequence, hindered adipogenesis reduce the fat storage capacity of adipose tissue. This results in ectopic lipid accumulation in liver and skeletal muscle leading to insulin resistance of these metabolically important tissues and eventually type 2 diabetes [9], [10].

In rodents, *B lymphocyte stimulator* (BLyS) has recently been demonstrated to be secreted by hypertrophic, mature adipocytes [11]–[13]. BLyS is known to play an important role in activating B-lymphocytes [14], [15]. Hence, BLyS is considered as a novel factor that links obesity to inflammation [11]. BLyS, also termed as B cell-activating factor (BAFF), belongs to the TNF ligand family, and has been identified as a factor that promotes B cell proliferation and immunoglobulin secretion [14], [15].

Since *in vivo* data in humans on the pathological significance of BLyS in low-grade inflammation of adipose tissue are rare, we intended to clarify whether (1) BLyS is expressed in human adipocytes *in vivo*, (2) BLyS expression in adipose tissue *in vivo* differs between lean and obese +/− insulin resistant human subjects, (3) BLyS serum concentrations are dysregulated in obese +/− insulin resistant patients, (4) BLyS responds to different weight loss therapies in obese humans and (5) inhibition of BLyS in humans *in vivo* by the neutralizing antibody belimumab alters insulin sensitivity.

## Materials and Methods

All studies were approved by the local ethics committees (Number: D475/11, University of Kiel, Germany). Written informed consent was obtained from each subject before inclusion into the study.

### Study populations

*1.) Adipose tissue biopsy study populations*. Human subcutaneous adipose tissue biopsies for immunohistology were taken during elective surgical procedures from lean controls (n = 3; 45.3+11.6 years; 66.7% males; body mass index (BMI): 23+1.3 kg/m^2^), overweight subjects (n = 3; 48.3+4.2 years; 66.7% males; 27.6+1.7 kg/m^2^) and obese individuals with type 2 diabetes (n = 3; 53.7+15.5 years; 33.3% males; 36.8+1.5 kg/m^2^). Exclusion criteria for this study have been described earlier [6]. Type 2 diabetes in patients of the third group was sufficiently controlled only by dietary intervention with or without metformin therapy.

For determining BLyS transcript levels in total adipose tissue samples, RNA was isolated from subcutaneous and visceral adipose tissue which was obtained during elective surgical procedures. The RNA study population consisted of lean controls and obese individuals with and without insulin resistance. Lean controls and grade 3 obese subjects +/− type 2 diabetes were equally distributed regarding gender while BMI and age were significantly different (**table 1**).

**Table 1:** Baseline characteristics of the study populations of the BLyS mRNA in human total adipose tissue biopsies of lean, grade 3 obese +/− insulin resistance (IR) and of BLyS measurements in sera of lean, grade 1+2 obese +/− insulin resistance.

mRNA level	*visceral*						
	lean	obese	obese+IR	p-value	t_1_	t_2_	t_3_
**male∶female**	**10∶6**	**19∶3**	**5∶1**	**0.2071**			
**BMI (kg/m^2^)**	22.1+2.9	52.9+9.2	50.4+7.2	<0.0001	<0.001	<0.001	ns
**age (years)**	57.1+5.2	42.7+10.4	47.7+9.0	<0.0001	<0.001	ns	ns

	*subcutaneous*						
	lean	obese	obese+IR	p-value	t_1_	t_2_	t_3_
**male∶female**	**11∶4**	**25∶2**	**7∶4**	**0.0776**			
**BMI (kg/m^2^)**	22.6+1.9	47.2+11.1	47.5+10.5	<0.0001	<0.001	<0.001	ns
**age (years)**	57.0+5.3	40.6+10.1	52.8+11.8	<0.0001	<0.001	ns	<0.01

serum level							
	lean	obese	obese+IR	p-value	t_1_	t_2_	t_3_
**male∶female**	**2∶8**	**2∶8**	**2∶8**	1.0000			
**age (years)**	57.7+14.66	56.4+10.7	57.3+4.4	0.9627			
**weight (kg)**	62.9+9.9	112.9+21.9	111.1+23.5	<0.0001	<0.001	<0.001	ns
**BMI (kg/m^2^)**	21.9+2.5	37.9+6.9	38.1+5.0	<0.0001	<0.001	<0.001	ns
**glucose (mg/dl)**	96.0+11.7	94.3+11.2	103.1+12.2	0.1539			
**insulin (µU/ml)**	5.1+2.5	7.2+1.3	27.8+8.1	<0.0001	ns	<0.001	<0.001
**triglycerides (mg/dl)**	91.5+39.3	119.4+57.1	150.8+58.0	0.0550			
**CRP (mg/l)**	1.1+0.4	4.4+3.8	7.3+8.3	0.0008	<0.01	<0.01	ns
**HOMA-IR index**	1.2+0.6	1.7+0.2	7.0+1.8	<0.0001	ns	<0.001	<0.001
**BLyS (pg/ml)**	543.7+63.9	514.1+140.9	600.2+168.6	0.1876			

t_1_: lean vs. obese.

t_2_: lean vs. obese+IR.

t_3_: obese vs. obese+IR.

Shown are means+SD for each group.

*2.) FoCus cohort study population*. As part of the Food Chain Plus (FoCus) project [http://www.focus.uni-kiel.de] a total number of n = 30 serum samples were obtained. The study population was divided into a lean control group, a grade 1+2 obesity group without insulin resistance and a grade 1+2 obesity group with insulin resistance (**table 1**).

*3.) Nutritional intervention study population*. The nutritional intervention group consisted of n = 16 subjects (45.3+7.4 years, 43.8% males, 42.2+6.2 kg/m^2^). This group was treated with a very-low-calorie formula diet (VLCD, Optifast 52 (Nestle Nutrition)) with a total caloric intake of approximately 800 kcal/d for 3 months. Inclusion criteria of this study population have been described earlier [16]. Seven patients suffered from arterial hypertension; one patient each suffered from gastroesophageal reflux, gonarthrose, non-alcoholic steatohepatitis or asthma. Two patients suffered from type 2 diabetes which was being controlled appropriately. One patient received a hormone replacement therapy; two patients took ß-blocker and one patient ACE inhibitors. Antihistamines or gastric acid neutralizing drugs were being taken by one patient each.

*4.) Bariatric surgery intervention study population*. Sera of 26 patients (42.1+11.8 years, 11.5% males, 48.7+7.4 kg/m^2^) were obtained before and 6 months after bariatric surgery. This intervention led to a mean weight reduction of 37.5 kg (140.7+28.8 kg to 103.2+25.4 kg). In the scope of bariatric surgery three patients received gastric bypass whereas all other subjects underwent sleeve gastrectomy. Regarding comorbidities and medications, seven patients suffered from arterial hypertension. One patient each had diabetic neuropathy, hyperuremia, epilepsy, gastroesophageal reflux or bronchial asthma. Thyroid and gallbladder have been removed in one patient. Two patients suffered from efficiently controlled hypothyroidism and three from type 2 diabetes which was controlled by insulin and metformin therapy. Three patients took calcium antagonists; six patients took ACE inhibitors and four patients ß-blocker. Diuretics were being taken by six patients. Nine patients regularly took analgetics; three patients cholesterol-reducing drugs. Nine patients took antidepressants, three patients were on allopurinol, 11 patients took gastric acid neutralizing drugs. Furthermore, four patients took thyroxin.

*5.) Belimumab intervention study population*. A total number of n = 5 patients with treatment indication for belimumab (Benlysta (Human Genome Sciences and GlaxoSmithKline), approved for the treatment of systemic lupus erythematodes), an anti-BLyS human monoclonal antibody, were treated for 4 weeks. 5 female patients with systemic lupus erythematodes (SLE) were included. BLyS is known to be elevated in patients with autoimmune diseases, such as *systemic lupus erythematosus* (SLE), Sjogren's syndrome, and rheumatoid arthritis [17]. One patient was additionally suffering from rheumatoid arthritis, and one patient had additionally pleuritis. Mean age was 48.4+18.9 years, mean BMI of these females was 26.6+4.3 kg/m^2^. All patients showed a response to belimumab treatment in terms of SLE disease activity indicated by improvement in at least one clinical sign (e. g. fatique) or a laboratory measure (e. g. increase in leucocytes). In order to examine the effect on insulin sensitivity the HOMA-IR and the leptin-to-adiponectin-ratio (LAR) were determined before and after treatment as described earlier [8].

### Separation of stromal vascular fractions and mature adipocytes from human adipose tissue biopsies

Subcutaneous adipose tissue samples were obtained from obese subjects undergoing elective open abdominal surgery in general anesthesia. All subjects fasted for 6 hrs prior to the operation. Adipose tissue biopsies were taken under sterile conditions and were transported into the laboratory in phosphate buffered saline. After dicing the tissue into 1–2-mm pieces, samples were digested in collagenase buffer (Hanks' balanced salt solution (PAA Laboratories GmbH, Cölbe, Germany), 3 mg/ml type I collagenase (Life Technologies GmbH, Darmstadt, Germany), and 2% bovine serum albumin (biomol GmbH, Hamburg, Germany) at 37°C for 1 h. The digest was filtered through a 260-µm stainless steel mesh and centrifuged at 400× *g* for 5 min. The supernatant containing the floating mature adipocytes was lysed in RIPA buffer (50 mM Tris-HCl, 150 mM NaCl, 0.5% sodium deoxycholate, 0.1% SDS, 1% NP-40, PhosSTOP (Roche, Penzberg, Germany), protease inhibitor cocktail (Sigma)). The cell pellet containing the stromal vascular fraction was treated with red cell lysis buffer (0.154 M NH4Cl, 10 mM KHCO3, 0.1 mM EDTA) for 5 min at room temperature. After several washing steps with red cell lysis buffer the pellet containing the stromal vascular fraction was lysed in RIPA buffer, PhosSTOP and protease inhibitor cocktail. Afterwards, lysed cell extracts were centrifuged at 4°C at 14,000 rpm for 30 min.

### Western Blotting

40 µg of protein per sample were added to 4× loading buffer (bromophenol blue and Laemmli buffer), heated to 95°C for 5 min, and separated on a 12% SDS-PAGE. Proteins were transferred to polyvinylidene fluoride membranes (Carl Roth GmbH, Karlsruhe, Germany). The following antibodies were used according to the instructions of the manufacturer: BLyS (ab16081; Abcam, USA), BCMA (ab5972, Abcam, USA). GAPDH (#2118, Cell Signaling, USA) and all secondary antibodies except anti-rat (ab102265, Abcam, USA) were purchased from Cell Signaling Technology (Frankfurt, Germany).

### RNA isolation and *Taqman* Quantitative Real Time Reverse Transcription-PCR

100 milligrams of frozen total adipose tissue from lean and grade 3 obese +/− type 2 diabetes were ground with 3 mm stainless steel balls using liquid nitrogen cooling (MM301 Mixer Mill, Retsch, Haan, Germany) for 30 sec to a fine powder. RNA was isolated using Trizol (Life Technologies GmbH) following the manufacturer's protocol. cDNA synthesis was further performed using Advantage RT for PCR kit (Clontech Laboratories, Saint-Germain-en-Laye, France) according to the manufacturer's instructions. Expression levels of BLyS and housekeeping gene ß-actin were determined using suitable TaqMan Gene Expression Assay kits (for BLyS: Hs00198106_m1; for ß-actin: Hs99999903_m1; Applied Biosystems, Darmstadt, Germany).

### Immunohistology

Immunohistochemistry was performed using BLyS antibody (LS-B2081; LifeSpan BioScience) as described earlier [16].

### Enzyme-linked immunosorbent assay (ELISA)

Serum was assayed for BLyS using the Quantikine ELISA Kit (DBLYS0B; R&D Systems, Inc., Wiesbaden, Germany) according to the manufacturer's instructions (according to the manufacturer: Sensitivity: 6.44 pg/ml. Specificity: <0.5% cross-reactivity observed with available related molecules. <50% cross-species reactivity observed with species tested). ELISA for adipokine measurement were purchased from IBL International (Hamburg, Germany) as follows: Adiponectin ELISA (BV51001, analytical-sensitivity: 0.2 µg/ml), Leptin ELISA (RE53151, analytical-sensitivity: 1.0 ng/ml) and were performed according to the instructions of the manufacturer.

### Statistical analysis

Statistical analysis was performed using GraphPad Prism 5.01 Software. Associations between categorical variables were assessed by Chi-squared test. If the assumption of normal distribution of the continuous data was not violated Student's t-test or ANOVA was used. In case of not normal distributed data the Mann-Whitney U test or Kruskal-Wallis test was applied. Moreover, regression analysis with BLyS expression as the dependent variable was used in order to avoid an effect of possible confounding factors as age. The significance level was set at 5% and multiple comparisons were done using Bonferroni or Dunn's post test.

## Results

### BLyS expression in human adipose tissue

**Figure 1:**
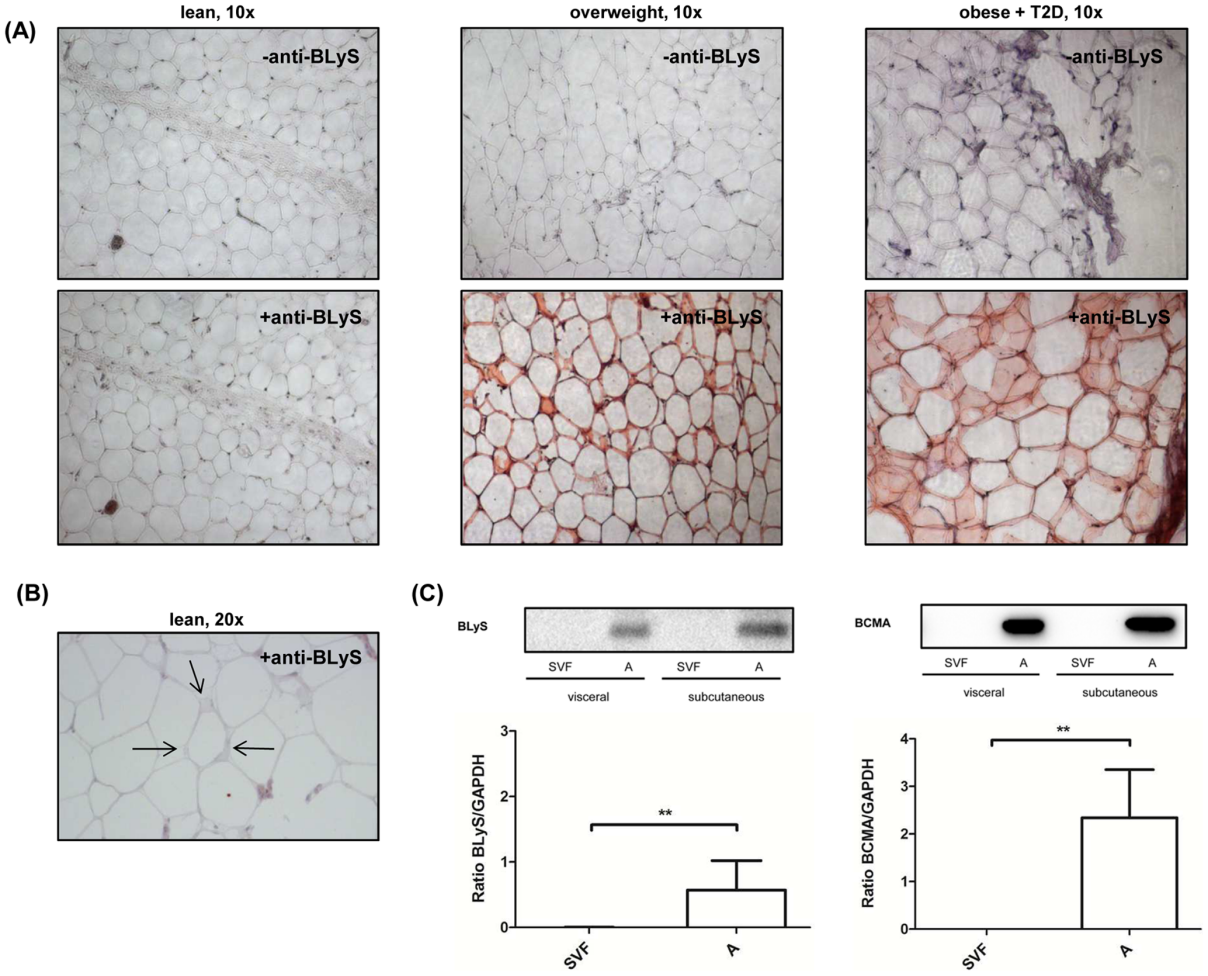
BLyS expression in adipose tissue in humans *in vivo*. (A) BLyS immunohistochemistry (lower panel) and haematoxylin staining (upper panel) of subcutaneous adipose tissue biopsies of lean (left), overweight (middle) and obese+type 2 diabetes (T2D) (right) human subjects (10×). The reddish color represents BLyS-positive mature adipocytes. (B) shows 20× magnification of the immunohistochemistry of a lean adipose tissue biopsy in which the arrows demonstrate the stromal vascular fraction. The lean representative was chosen as BLyS expression was rarely detectable in their adipocytes, thus resulting in a more reliable visual interpretation of the stromal vascular fraction. (C) BLyS and BCMA protein detection revealed that BLyS and BCMA is only expressed in the mature adipocyte [A] fraction but not in the stromal vascular fraction [SVF]. Densitometry graphs data in this figure are shown as means+SEM (n = 5).

Immunohistochemistry of adipose tissue biopsies exhibited BLyS to be present within the cytoplasm of adipocytes *in vivo* in human subjects with obesity with and without type 2 diabetes but to a lesser extend in lean controls (**figure 1A**). The higher magnification shown in **figure 1B** suggests that the major source of BLyS within adipose tissue are mature adipocytes, since no obvious staining was observed in the stromal vascular fraction. In order to further examine whether BLyS is only expressed in mature adipocytes or also in certain cells of the stromal vascular fraction, we next separated mature adipocytes and the stromal vascular fraction of visceral and subcutaneous adipose tissue biopsies from n = 5 obese humans by collagenase digestion and performed western blots for BLyS and the BLyS receptor BCMA. This experiment showed that the major source of BLyS and BCMA in adipose tissue are mature adipocytes (**figure 1C**) while the different cells in the stromal vascular fraction do not contribute to adipose tissue BLyS and BCMA expression in a significant manner. Also, as shown in figure 1C, no significant difference in BLyS expression was found between subcutaneous and visceral mature adipocytes (**figure 1C**).

**Figure 2:**
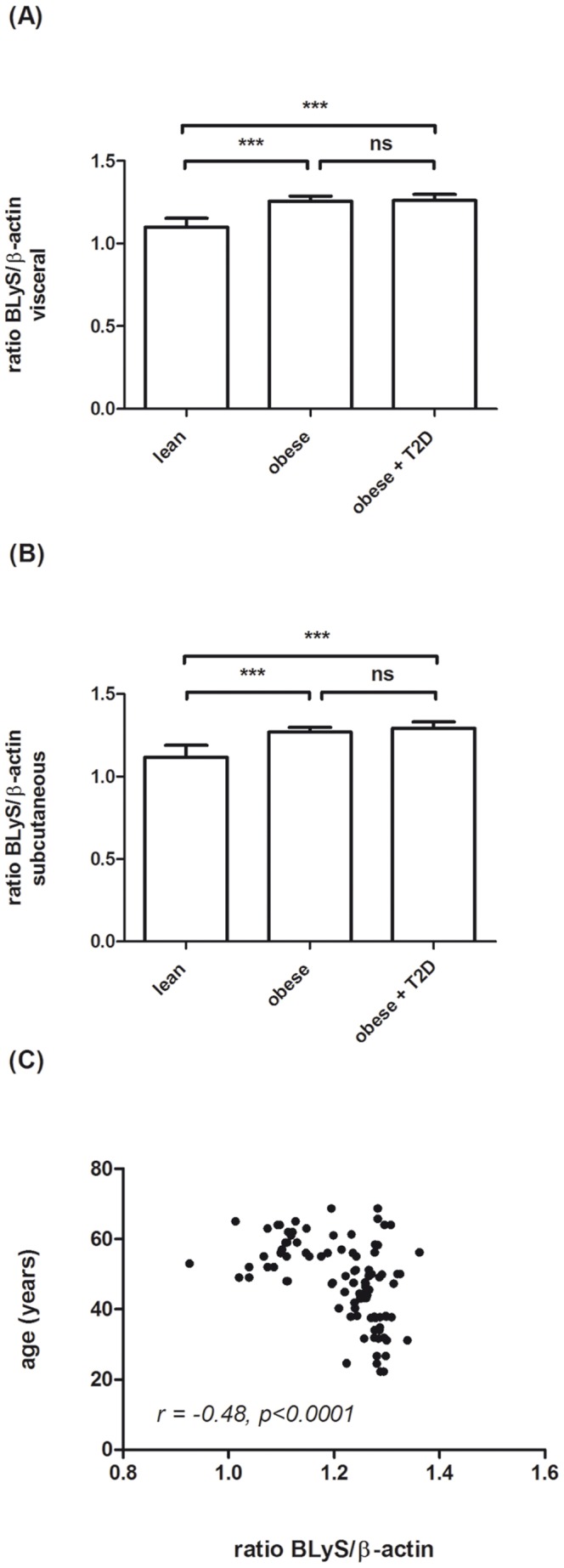
BLyS mRNA level in (A) visceral and (B) subcutaneous adipose tissue of lean control subjects, patients with grade 3 obesity +/− type 2 diabetes (T2D). RNA was isolated from total subcutaneous and visceral adipose tissue biopsies. Data in this figure are shown as means+SD. (C) shows a significant negative correlation between age and BLyS expression (*r* = −0.48, p<0.0001) in a combined analysis of all biopsy samples.

In order to compare BLyS expression levels between lean controls and obese human subjects with and without type 2 diabetes, we performed real-time RT-PCR using RNA extracted from human visceral and subcutaneous adipose tissue biopsies. These experiments revealed a significant increase in BLyS expression in both adipose tissue depots of obese human subjects compared to lean controls (**figure 2A+B**). However, as shown in **figure 2A+B**, BLyS expression levels did not significantly differ between non-diabetic and diabetic obese human subjects. Further statistical analysis showed that relative BLyS expression was higher in males (n = 77) than in females (n = 20) (1.23+0.09 versus 1.18+0.08, p<0.05), higher in subcutaneous (n = 53) than in visceral (n = 44) adipose tissue (1.23+0.09 versus 1.20+0.09, p<0.05) and higher in diabetic (n = 17) than in non-diabetic (n = 80) subjects (1.28+0.04 versus 1.20+0.09, p<0.01). In addition we found a significant negative correlation with age (*r* = −0.48, p<0.0001 (**figure 2**)). Since the mean age was different between the groups, we also performed age-adjusted analysis of the expression levels shown in figure 2. This analysis revealed stable results, indicating that age is not a major confounder of the results obtained.

### BLyS serum concentrations in human obesity

In order to investigate whether BLyS serum levels vary between lean controls, grade 1+2 obese individuals (BMI 30–40 kg/m^2^) with insulin resistance and grade 1+2 obese individuals (BMI 30–40 kg/m^2^) without insulin resistance, we performed ELISA assays in serum samples collected from such subjects. The groups were equally distributed in terms of age and gender (**table 1**). Insulin resistance was estimated by the HOMA-IR index for which values below 2.0 were supposed to be as normal [18]. As shown in **table 1**, in this analysis BLyS serum concentrations ranged from 543.7+63.9 pg/ml to 600.2+168.6 pg/ml and were not significantly different between the groups.

**Figure 3:**
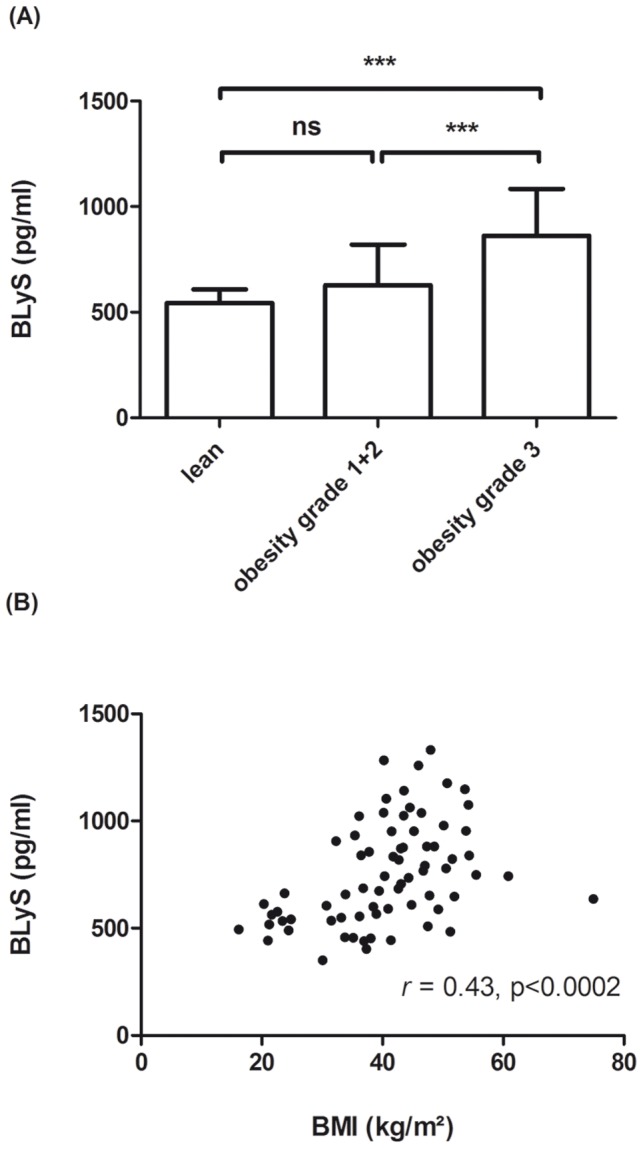
BLyS serum levels in human obesity. (A) represents significant differences between different grades of obesity (grade 1+2 (BMI≥30 to <40 kg/m^2^), n = 20 and grade 3 (BMI≥40 kg/m^2^), n = 42) and normal-weight ((BMI≤25 kg/m^2^), n = 10) human control subjects. (B) shows the correlation (*r* = 0.43, p<0.0002) between BLyS serum levels and BMI of n = 72 human subjects used in this study. Data in this figure are shown as means+SD.

Since we did not find a significant association of BLyS serum levels with grade 1 and 2 obesity (BMI 30–40 kg/m^2^), we next aimed to examine whether BLyS serum levels are increased in grade 3 obese subjects (BMI>40 kg/m^2^). This experiment showed that serum BLyS concentrations are elevated in grade 3 obesity (mean BMI: 47.5+6.6 kg/m^2^, mean BLyS: 862.5+222.0 pg/ml) which was significantly different to lean controls (mean BMI: 21.9+2.5 kg/m^2^, mean BLyS: 543.7+60.7 pg/ml) and grade 1+2 obese subjects (mean BMI: 35.4+2.8 kg/m^2^, mean BLyS: 627.6+193.2 pg/ml) (**figure 3A**). Correlation analysis of serum BLyS levels with the BMI in the different subjects of our research cohorts (n = 72) indicated an overall significant positive correlation (*r* = 0.43, p<0.0002) (**figure 3B**).

### Effects of VLCD and bariatric surgery on BLyS serum levels

**Figure 4:**
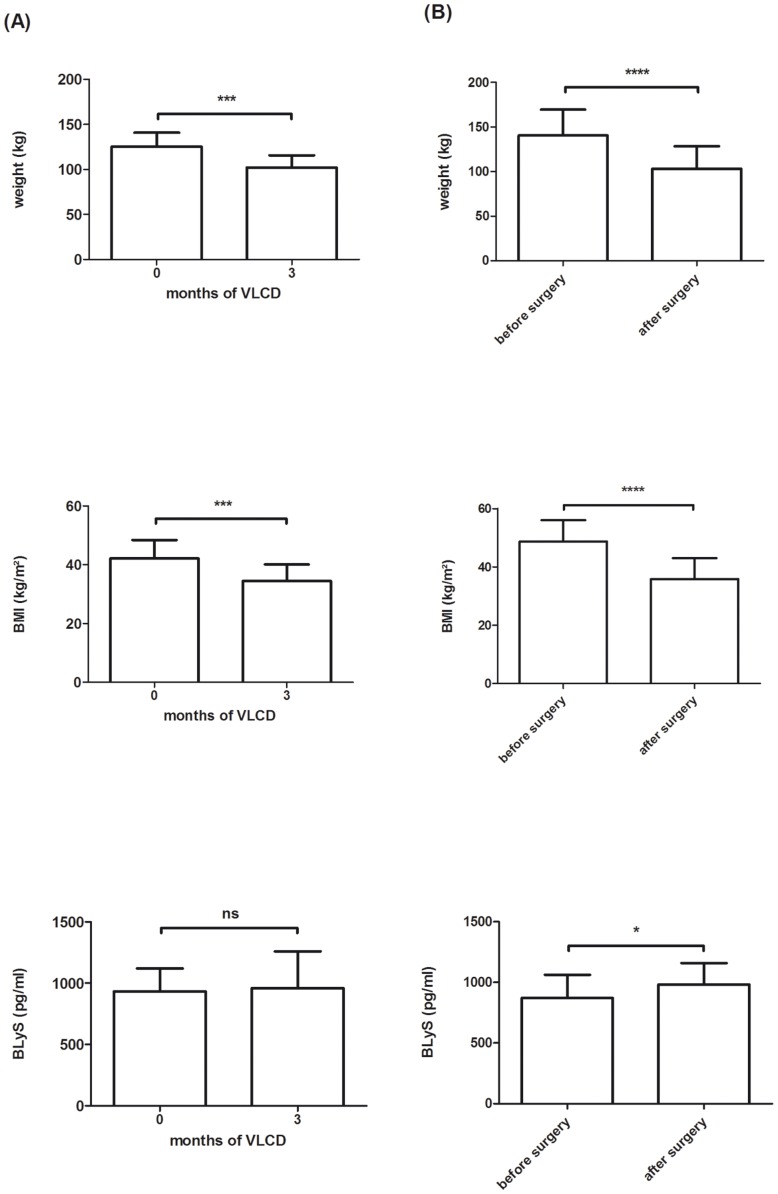
Anthropometric measures and BLyS serum concentrations before and after (3 months) of a very low calorie formula diet (VLCD) (approximately 800 kcal/d) as well as before and after (6 months) bariatric surgery in obese individuals. (A) Significant weight reduction (upper and middle panel) and no change of BLyS serum levels by VLCD (lower panel). Data in this figure are shown as means+SD of n = 16 obese human subjects during a VLCD. (B) Significant weight reduction (upper and middle panel) and significant increase of BLyS serum levels by bariatric surgery (lower panel). Shown are means+SD for n = 26 patients.

As there is evidence that weight-loss strategies reduce low-grade inflammation in obese human subjects [16], [19], we next examined BLyS serum levels before and after different weight loss strategies. In the first study population, 16 obese subjects were treated with a nutritional intervention using a VLCD (approximately 800 kcal/d) for 3 months. Mean weight loss achieved accounted for 17.9 kg (125.2+15.5 kg to 102.5+13.2 kg) corresponding to a BMI reduction from 42.2+6.2 kg/m^2^ to 34.6+5.6 kg/m^2^. BLyS serum levels were not reduced due to the weight loss achieved and accounted to 934.22+185.8 pg/ml before and 960.72+300.4 pg/ml after 3 months of the nutritional intervention (**figure 4A**). In the second study population, BLyS serum samples were measured in obese human subjects before and 6 months after bariatric surgery. The later time point for blood sampling in the surgical group (6 months versus 3 months in the VLCD) was used, since several weeks after a surgical procedure is performed healing processes including inflammatory reactions can be expected which potentially will also induced BLyS expression. Bariatric surgery induced a mean weight loss of 37.5 kg (140.7+28.8 kg to 103.2+25.4 kg) in a time period of 6 months (BMI before: 48.7+7.4 kg/m^2^; BMI after: 35.7+7.2 kg/m^2^). Interestingly, as for what was found for the VLCD, BLyS serum levels were not reduced after the surgical intervention. Instead BLyS levels exhibited even a mild but significant increase (BLyS before: 872.5+189.1 pg/ml; BLyS after: 982.0+177.1 pg/ml, p = 0.03) (**figure 4B**).

### Effects of BLyS inhibition on insulin sensitivity in human subjects *in vivo*

In order to examine whether BLyS is of functional relevance in terms of development of insulin resistance in humans *in vivo*, the effect of BLyS on human insulin sensitivity was examined in a translational research approach. Therefore, blood samples were drawn before and 4 weeks after treatment of human subjects with the neutralizing monoclonal anti-BLyS antibody belimumab. Subsequently, insulin sensitivity was estimated by HOMA-IR and the leptin-to-adiponectin ratio (LAR). LAR is a very accurate measure for insulin sensitivity with close correlation to hyperinsulinemic-euglycemic clamp data [18]. The study population exhibited reduced insulin sensitivity before administration of belimumab, as indicated by an increased basal HOMA-IR and LAR. As shown in **table 2**, neither HOMA-IR nor LAR was influenced by belimumab treatment.

**Table 2:** Insulin resistance before and after belimumab treatment.

	before	after	p-value
insulin (µU/ml)	23.4+19.3	21.5+20.0	0.8857
glucose (mg/dl)	96.5+18.4	85.5+11.1	0.3429
**HOMA-IR index**	**5.1+3.5**	**4.3+3.7**	**0.4857**
leptin	24.8+8.4	26.8+14.9	0.8031
adiponectin	19.1+10.2	16.2+3–9	0.6180
**LAR**	**1.4+0.6**	**1.3+0.5**	**0.9687**

Insulin resistance was measured by HOMA-IR and leptin-to-adiponectin-ratio (LAR). Data are given as means+SD of n = 5 patients treated with anti-BLyS antibody belimumab for a time period of 4 weeks.

## Discussion

Adipose tissue has long been regarded as a silent and passive organ, storing excess energy as triglycerides and releasing energy as fatty acids. Due to intensive research over the last decades adipose tissue now is also referred to as an endocrine and immune organ secreting a wide variety of metabolic and immune factors. They can initiate and influence inflammation, metabolism, appetite regulation and many other processes [20], [21].

BLyS is a TNF ligand family member known to promote B-cell proliferation and immunoglobulin secretion [22], [23]. In the present study, we demonstrate that within human adipose tissue BLyS is expressed in mature adipocytes and that expression is increased in obesity in both, visceral and subcutaneous fat depots (**Figure 2**). Further analysis showed that mature adipocytes are the major source of BLyS within adipose tissue, since we did not find a significant expression in the stromal vascular fraction (**figure 1**). This finding also suggests that BLyS is up-regulated during differentiation of preadipocytes (localized within stromal vascular fraction) into mature fat cells, which is in agreement to previously reported findings in the murine 3T3-L1 cell line [11], [12].

Significant higher serum levels were found in grade 3 obese patients (BMI≥40 kg/m^2^) but not in grade 1+2 obese subjects (**figure 3**) compared to lean controls. In this respect it has to be mentioned, that most factors identified so far in low-grade inflammation of adipose tissue are part of the innate immune system [5]. BLyS on the other hand is known to activate B-lymphocytes and to promote immunoglobulin secretion and thereby serves a marker for the adaptive immune system. Hence, the data presented here suggest that both compartments of the immune system are activated in human obesity, which is in agreement with several recent reports. For example Fabbrini E et al. in 2013 showed an association between adipose tissue CD4^+^ T-cell populations and insulin resistance in obese human individuals [24].

Factors of the innate immunity induced in low-grade inflammation of adipose tissue are known to be involved in the pathogenesis of insulin resistance. For example, in one of our previous studies we have shown that blockade of IL-6 signaling in humans *in vivo* by the monoclonal antibody tocilizumab increased insulin sensitivity [10]. Using a similar translational approach, in the present study we examined insulin sensitivity in humans treated with belimumab, a monoclonal antibody blocking BLyS activity *in vivo*. In contrast to what was shown for IL-6 inhibition, in that experiment BLyS inhibition did not significantly alter insulin sensitivity (**table 2**). It has to be mentioned that this experiment has to be interpreted carefully, since only 5 human individuals have been examined and all of the subjects were suffering from an immunological disease. However, this finding is in agreement with the data, that in the present study neither BLyS expression levels in human adipose tissue nor BLyS serum levels in obese human subjects are associated with insulin resistance (**figure 2** **,** **table 1**).

The data obtained in the present study may even suggest that BLyS might display potential beneficial immune-modulating effects, since we identified BLyS not to be reduced but up-regulated after bariatric surgery of grade 3 obese subjects (**figure 4B**). Also, a weight loss to a lower extend by a VLCD did not significantly reduce BLyS serum concentrations in the present study (**figure 4A**). This is in contrast to was found for factors of the innate immunity. For example TNF-α and IL-6 have both been shown to be down-regulated during weight loss procedures [25], [26].

The hypothesis that BLyS might be a beneficial factor in metabolic inflammation in human obesity is further supported by the data of an independent research group, showing that BLyS expression, like PPAR-γ expression, is induced during 3T3-L1 adipogenesis [13], supposing a positive but not a negative effect in human adipose tissue biology. Thus, taking these findings together it might be speculated that BLyS serves as a balancing immune modulator rather than a classical pro-inflammatory factor in low-grade inflammation of adipose tissue. On the other hand, since the status of B-cells in human adipose tissue at the present time is not so clear as in animal models, BLyS could also play solely a role in adipocyte differentiation rather than playing a potent link with adaptive immunity.

In summary, the present study provides evidence, that BLyS is a novel factor in human adipose tissue biology. Thereby BLyS exerts several important differences compared to most other factors in that field: (1) BLyS is specifically related to grade 3 obesity but not to grade 1+2 obesity, (2) BLyS links the adaptive rather than innate immune system to metabolism, (3) BLyS is not involved in the pathogenesis of human insulin resistance and (4) the increase of BLyS after bariatric surgery might indicate beneficial immune-modulating activity rather than negative effects on adipose tissue biology. Therefore, BLyS will be an interesting research candidate for future studies in the growing field of metabolic inflammation.
